# Erector spinae plane block versus intercostal nerve block for postoperative analgesia in lung cancer surgery

**DOI:** 10.2478/raon-2023-0035

**Published:** 2023-09-04

**Authors:** Polona Gams, Marko Bitenc, Nenad Danojevic, Tomaz Jensterle, Aleksander Sadikov, Vida Groznik, Maja Sostaric

**Affiliations:** Surgery Bitenc, Thoracic Surgery Clinic, Golnik, Slovenia; Faculty of Medicine, University of Ljubljana, Ljubljana, Slovenia; Faculty of Computer and Information Science, University of Ljubljana, Ljubljana, Slovenia; Faculty of Mathematics, Natural Sciences and Information Technologies, University of Primorska, Koper, Slovenia; University Medical Center Ljubljana, Ljubljana, Slovenia

**Keywords:** erector spinae plane block, intercostal nerve block, postoperative analgesia, video-assisted thoracic surgery, thoracic anesthesia

## Abstract

**Background:**

A recent trend in postoperative analgesia for lung cancer surgery relies on regional nerve blocks with decreased opioid administration. Our study aims to critically assess the continuous ultrasound-guided *erector spinae* plane block (ESPB) at our institution and compare it to a standard regional anesthetic technique, the intercostal nerve block (ICNB).

**Patients and methods:**

A prospective randomized-control study was performed to compare outcomes of patients, scheduled for video-assisted thoracoscopic (VATS) lung cancer resection, allocated to the ESPB or ICNB group. Primary outcomes were total opioid consumption and subjective pain scores at rest and cough each hour in 48 h after surgery. The secondary outcome was respiratory muscle strength, measured by maximal inspiratory and expiratory pressures (MIP/MEP) after 24 h and 48 h.

**Results:**

60 patients met the inclusion criteria, half ESPB. Total opioid consumption in the first 48 h was 21. 64 ± 14.22 mg in the ESPB group and 38.34 ± 29.91 mg in the ICNB group (p = 0.035). The patients in the ESPB group had lower numerical rating scores at rest than in the ICNB group (1.19 ± 0.73 *vs.* 1.77 ± 1.01, p = 0.039). There were no significant differences in MIP/MEP decrease from baseline after 24 h (MIP p = 0.088, MEP p = 0.182) or 48 h (MIP p = 0.110, MEP p = 0.645), time to chest tube removal or hospital discharge between the two groups.

**Conclusions:**

In the first 48 h after surgery, patients with continuous ESPB required fewer opioids and reported less pain than patients with ICNB. There were no differences regarding respiratory muscle strength, postoperative complications, and time to hospital discharge. In addition, continuous ESPB demanded more surveillance than ICNB.

## Introduction

Post-operative analgesia is crucial for early rehabilitation in thoracic surgery, as patients are required to actively participate in respiratory physiotherapy.^1,2^ Uncontrolled pain requires high doses of opioid analgesics which should be avoided according to the Early Recovery After Surgery (ERAS) guidelines.^3^ An opioid-sparing analgetic regimen is used to alleviate their numerous side effects, such as nausea, vomiting, constipation, lethargy, and respiratory depression.^4^ Regional techniques have been implemented to reduce the need for opioid analgesics.^5,6^

In an attempt to find safe and less invasive methods of postoperative analgesia, new techniques of nerve blocks have emerged. Among other peripheral blocks, intercostal nerve block (ICNB) and *erector spinae* plane block (ESPB) are being introduced in recent years.^7,8^ In comparison to the neuraxial blockade, they have a lower incidence of spinal cord injury, epidural hematoma, and central nervous system infection.^9,10,11^

The ICNB applied intrathoracically at the end of the surgery, is a regional technique currently used for video-assisted thoracoscopic (VATS) procedures at our surgical center. While fairly simple to use and applied under direct vision, there are some disadvantages of the ICNB: limited time of analgesic effect, which cannot be extended by a continuous infusion, application at the end of surgery instead of pre-incision and multiple injections that are needed to pertain a single block. The block cannot be executed in the presence of pleural infection, e.g., empyema.^12,13,14^

The *erector spinae* plane block (ESPB) was first described by Forero *et al*. in 2016 as a thoracolumbar interfascial plane block for treating severe neuropathic pain from the ribs.^15^ This interfascial nerve block is applied under ultrasound guidance and has a large safety margin. It can also be applied to patients on anticoagulant drugs. Cadaveric studies pointed out that the local anesthetic spreads to the thoracic paravertebral space^16^ or epidural space^17^, whereas some stated that it spreads more lateral on the thoracic wall without passing the costotransverse foramen.^18^ Its efficacy for thoracic surgeries in the first 24 hours after surgery as a single shot has been proven in previous studies and meta-analyses.^19,20,21^

We compared the continuous ultrasound-guided ESPB with ICNB to evaluate their analgetic efficacy in patients after lung cancer resection, representing our institution's first study of continuous ESPB in Slovenia. We present the following article in accordance with the CONSORT reporting checklist.

## Patients and methods

The study was approved by the National Ethics Committee under the number 0120-372/2019/7 and the study was registered to the Clinical Trial Registry under number NCT04665531.

### Patients

Sixty participants were enrolled between the 19^th^ of February 2020 and the 14^th^ of March 2022. Eligible patients with early-stage lung cancer were scheduled for VATS tumor resection with a three-port approach. 80% had lung lobe resection, 12% had marginal lung resection, and 8% had segmentectomy. Most of the patients had lung adenocarcinoma (72%), followed by epidermoid or squamous cell carcinoma (20%), small-cell lung carcinoma (2%), and metastasis (2%). Two tumors turned out to be benign.

Other inclusion criteria were ASA status I–III and informed written consent for participation in the study. Exclusion criteria were chronic pain syndrome, chronic opioid use, weight less than 50 kg due to risk of local anesthetic systemic toxicity (LAST), body mass index (BMI) > 35, pregnancy or breastfeeding, allergy to local anesthetics, inflammation at the catheter insertion site or inability to use the patient-controlled analgesia (PCA) pump. Patients were randomly assigned to either the intervention ESPB arm or the comparative ICNB arm. The study protocol could not be blinded because of the indiscrete intervention type.

### Nerve block technique

Patients in the ICNB group received a single-shot intrathoracic ICNB after tumor extraction, approximately 30 minutes before the end of the surgery. They received 20 ml of 0.5% levobupivacaine with injections at 6 intercostal spaces, adjacent to the surgical wound. The perineural intercostal space was located under direct vision. The same surgeon performed all the surgeries and executed the ICNBs.

Patients in the ESPB group received a 20 ml bolus of 0.5% levobupivacaine through the ESPB catheter approximately 30 minutes before the end of the surgery, continued by an infusion of 5 ml/h 0.2% ropivacaine with intermittent boluses of 15 ml per 4 hours.

**FIGURE 1.**
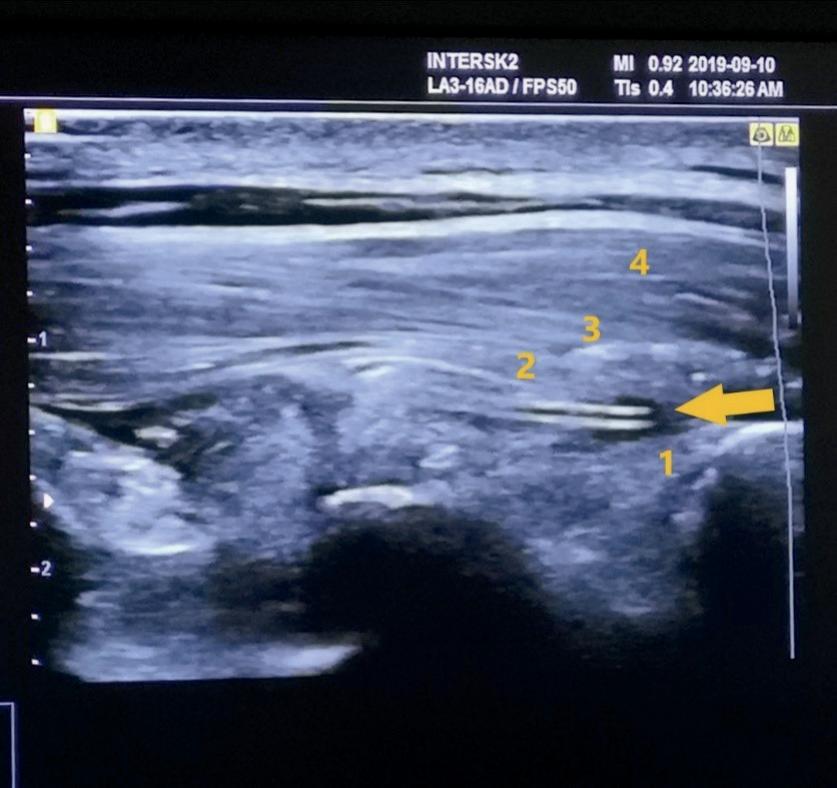
Ultrasound image of the inserted ESPB catheter (marked by an arrow) and interfascial hydro-dissection. 1 – underlying thoracic lamina, 2 – m. *erector spinae*, 3 – *m. rhomboideus*, 4 – *m. trapezius*

Two anesthesiologists, experienced in regional anesthesia, were inserting catheters to the patients before the surgery in the pre-op area. The standard monitoring and i.v. canal were applied before the intervention. The ESPB catheter insertion was performed using Samsung© ultrasound with a GE 12L-RS high-frequency linear probe. Aseptic conditions were guaranteed by using sterile drapes, sterile probe dressings, gloves, masks, and surgical gowns. The catheter insertion underwent in a pronated position with the patient lightly sedated by 1–2 mcg/kg fentanyl. The insertion site was infiltrated with 2 ml of 2% lidocaine on the T4 level of the spine, approximately 3 cm ipsilateral from the midline on the transverse process. The needle was then inserted under ultrasound guidance, positioning the needle tip immediately above the periosteum. The position was confirmed by injecting approximately 10 ml of 0.9% sodium chloride solution, which caused a hydro-dissection between the *erector spinae* muscle and the underlying fascia. Then, the catheter was inserted 4–6 cm above the needle tip. (Figure 1). After needle extraction, the catheter was secured using Stat-Lock© and multiple see-through coverings (Tegaderm©). Anesthesiologists used an 18G, 80 mm BD Microlance© pointed needle and a 20G Braun© multi-orifice epidural catheter.

### Peri-operative protocol

All the patients underwent the same anesthesia protocol. Induction to general anesthesia was conducted with 1.5–2 mg/kg propofol 1% and 0.75–1 mcg/kg remifentanil in a slow bolus followed by 0.6 mg/kg rocuronium. Total intravenous anesthesia was maintained with an infusion of 5 mg/kg/h propofol 1% and 0.02–0.03 mcg/kg/min remifentanil. Blood pressure was maintained with fluid administration and appropriate vasoactive drugs when needed. Reversion of neuromuscular block was performed by a bolus of 2 mg/kg sugammadex at the end of the surgery.

Patients were constantly monitored (ECG, pulse oximetry, invasive blood pressure) from admission to the pre-op area until at least 48 hours after surgery. Nurses at the intensive care unit documented the post-operative numerical rating scale for pain (NRS) every hour in the first 48 hours except when the patient was asleep. The patients expressed their current NRS on a scale from 0 to 10 with 0 meaning no pain and 10 meaning the worst pain imaginable. Nurses asked the patients about their NRS at rest and at cough in the last hour, which included spontaneous and active cough but not respiratory physiotherapy.

Every patient received a PCA pump with the protocol: demanded bolus 3 mg piritramide 1 mg/ml per 15 min with a maximum of 2 boluses per hour with no continuous infusion. If the pain reported by a patient was still higher than 3/10, a nurse applied an additional bolus of 3 mg piritramide. When a patient demanded more than 4 boluses per hour, the attending doctor initiated an infusion of 2 mg/h piritramide until the pain settled below 3/10. All the patients received regular doses of diclofenac and metamizole in terms of multimodal analgesia.

The postoperative care of the ESPB catheter followed the same protocol as for the thoracic-epidural catheter. The attending physician evaluated the catheter insertion site every day, looking for signs of infection.

All the patients performed maximal inspiratory and expiratory pressure (MIP/MEP) tests three times: on the day of the surgery prior to the surgical procedure, 24 and 48 hours later.

### Statistical analysis

The statistical analyses were performed using IBM SPSS version 25, Orange data mining and visualization suite.^22^ The patient and treatment characteristics were described using descriptive statistics. Demographic variables were compared with Pearson's Chi-square test. Cumulative piritramide use and NRS values are expressed as means with standard deviation. The potential differences between arms were assessed using the Kruskal-Wallis test. All the reported p-values are two-tailed with a significance level *α* < 0.05. The preoperative MIP and MEP measurements were set as baseline (100%), while the following measurements from the same patient were expressed as percentages from the baseline. Group medians were compared with the Student's t-test. Time to chest tube removal and hospital discharge were assessed by the median test.

## Results

**FIGURE 2.**
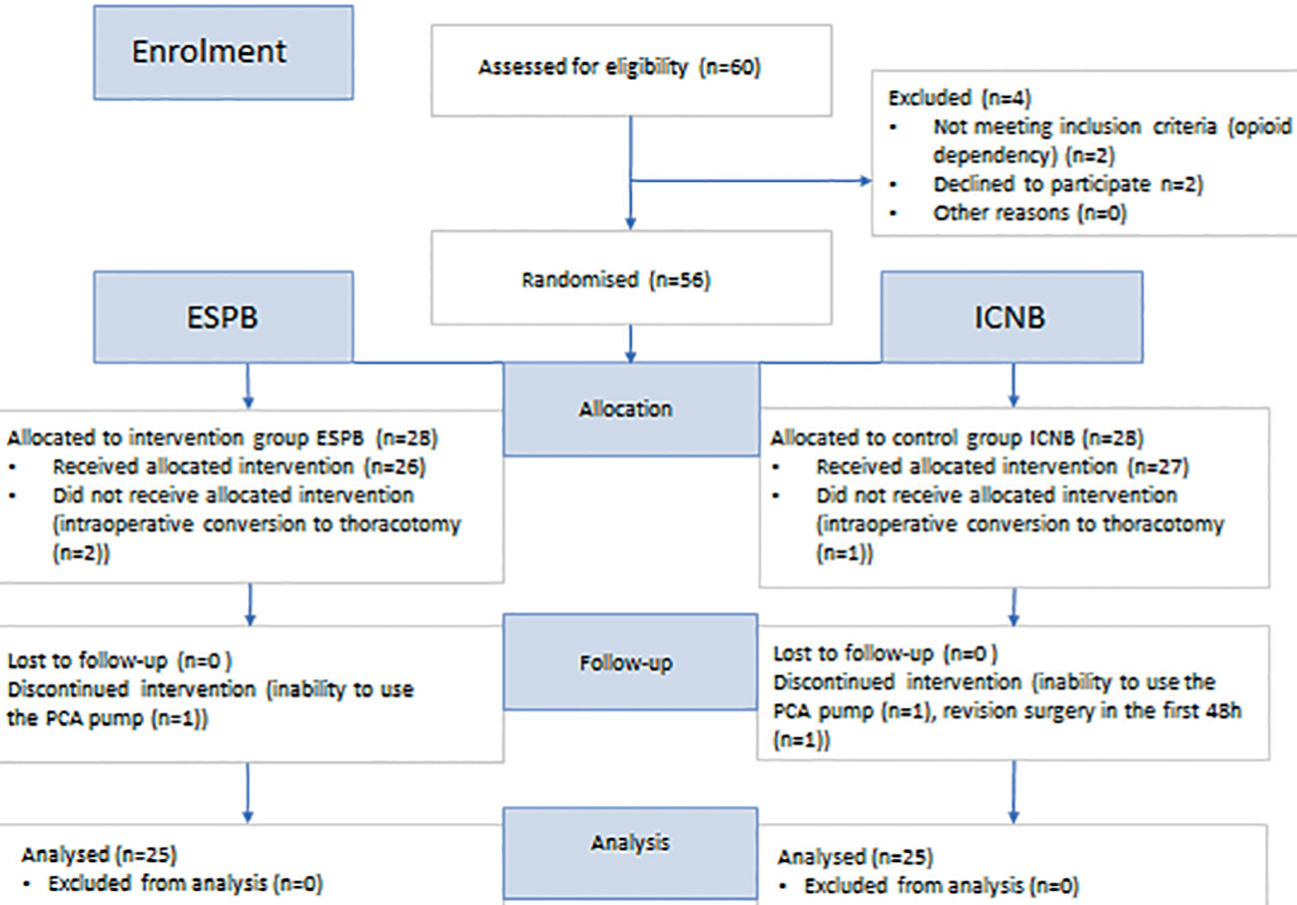
CONSORT flow diagram. ESPB *= erector spinae* plane block; ICNB = intercostal nerve block; PCA pump = patient-controlled analgesia pump

Patient allocation and follow-up are shown in the Consolidated Standards of Reporting Trials (CONSORT) flow diagram (Figure 2).

There were no statistically significant demographic differences between the study groups considering gender, age, body mass index, type of surgery, ASA and forced expiratory volume in the first second (FEV1) (Table 1).

**TABLE 1.** Demographic data

	**Total**	**ESPB**	**ICNB**	**p-value**
Patients enrolled	50	25	25	
Gender (male/female)	30/20	17/8	13/12	0.25
Age (years)^*^	69.80 ± 8.41	69.44 ± 8.20	70.2 ± 8.76	0.53
Height (cm)^*^	170.20 ± 8.49	172.04 ± 8.01	168.36 ± 8.72	0.15
Weight (kg)^*^	76.62 ± 14.07	76.24 ± 14.36	77.00 ± 14.03	0.72
BMI (kg/m^2^)^*^	26.37 ± 3.93	25.72 ± 4.18	27.03 ± 3.64	0.21
ASA 1/2/3	1/25/24	0/14/11	1/11/13	0.47
FEV1 before the surgery	94.78 ± 21.57	95.45 ± 21.84	94.17 ±21.77	0.88

1 Values under age, height, weight, and BMI are given as mean and 95% confidence interval.

ASA = American Society of Anesthesiologists assessment; BMI = body mass index; ESPB = *erector spinae* plane block; FEV1 = forced expiratory volume in the first second; ICNB = intercostal block

**FIGURE 3.**
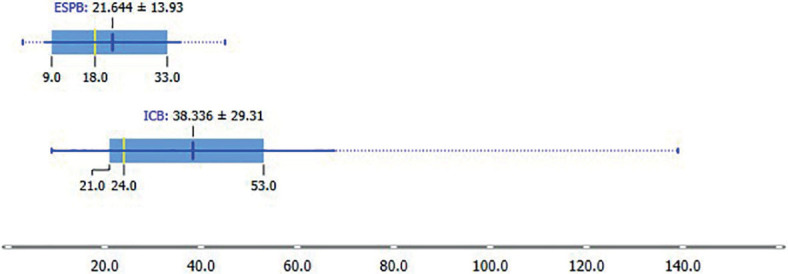
Cumulative piritramide use in the first 48 hours after surgery. The values in the graph are marked as mean (blue), median (yellow), and interquartile range. ESPB *= erector spinae* plane block; ICNB = intercostal nerve block

**FIGURE 4.**
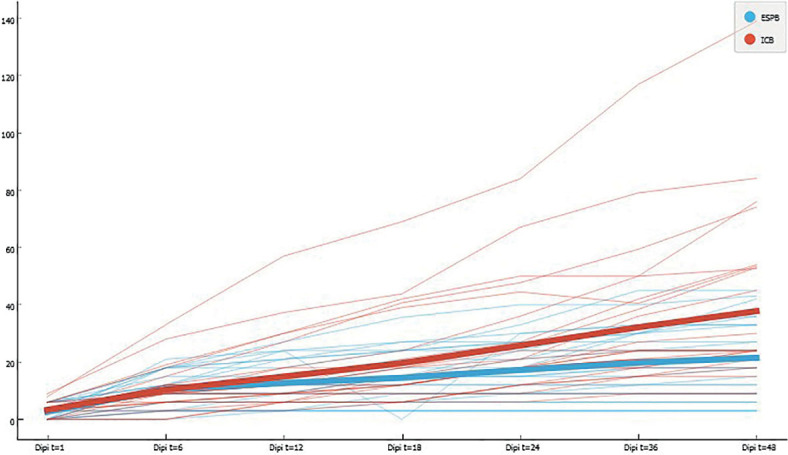
Cumulative opioid consumption in the first 48 h after surgery. The bold line shows the median values for each group.

The cumulative piritramide use in the first 48 hours after surgery was 21.64 ± 14.22 mg in the ESPB group and 38.34 ± 29.91 mg in the ICNB group (p = 0.035) (Figure 3). Figure 4 shows the cumulative linear graph of piritramide use in time for each group.

The mean NRS scores at rest in the first 48 hours after surgery were 1.19 ± 0.73 in the ESPB group and 1.77 ± 1.01 in the ICNB group. The difference between groups is statistically significant (p = 0.039). The mean NRS scores at cough in the first 48 hours after surgery were 2.53 ± 1.23 for ESPB and 2.85 ± 0.98 for ICNB. The difference between groups is statistically insignificant (p = 0.432).

There were no statistically significant differences between the ESPB and ICNB groups in MIP or MEP after 24 or 48 hours (Table 2).

**TABLE 2.** Maximal inspiratory and expiratory muscle strength

**Respiratory test values (%)**		**ESPB**	**ICNB**	**p-value**
MIP	24 hours	71.58 ± 16.69	75.98 ± 24.04	0.088
	48 hours	73.42 ± 19.10	88.11 ±30.72	0.110
MEP	24 hours	73.36 ± 20.82	85.55 ± 37.35	0.182
	48 hours	74.90 ± 22.39	98.90 ± 32.29	0.645

Respiratory test values are expressed as percentages from the baseline value.

ESPB *= erector spinae* plane block; ICNB = intercostal nerve block; MEP = maximal expiratory pressure; MIP = maximal inspiratory pressure

Time to chest tube removal was 4.13 ± 2.92 days in the ESPB group *vs.* 3.88 ± 2.26 days in the ICNB group (p = 0.41). Time to hospital discharge was 4.43 ± 2.87 days in the ESPB group *vs*. 4.08 ± 2.21 days in the ICNB group (p = 0.32).

There were no catheter-related complications such as clogging of the catheter, unintentional removal, or insertion-site infections. Attending physicians noted a few cases of minimal bleeding under the see-through coverings.

In the ESPB group, they noted one case of paroxysmal atrial fibrillation, one case of paroxysmal supraventricular tachycardia, and one case of sinus tachycardia at the time of observation. In the ICNB group, they noted 6 cases of paroxysmal atrial fibrillation. These arrhythmias emerged on postoperative day 1 or 2.^23^ There were no other acute or sub-acute complications related to regional anesthesia.

## Discussion

This study of postoperative analgesia for VATS lung cancer resection, comparing continuous ultrasound-guided ESPB versus ICNB, is the first of its kind in Slovenia. Due to the Covid-19 pandemic crisis, causing limited resources and additional healthcare concerns, the time of patient recruitment was prolonged, and only 60 patients from 200 were eligible for our study in a two-year period. The most significant findings were lower opioid demands in the ESPB group (21.64 ± 14.22 mg *vs*. 38.34 ± 29.91 mg (p = 0.035)) and lower cumulative NRS scores at rest in the first 48 hours after surgery (1.19 ± 0.73 *vs.* 1.77 ± 1.01, p = 0.039)) than in the ICNB group.

In recent years, some studies were comparing single-shot ICNB and ESPB with unclear advantages. In terms of postoperative opioid use, the ICNB was reported by some studies to be more efficient and by other studies to be less efficient than the ESPB.^24,25^ A previous pilot study demonstrated the feasibility of conducting a randomized controlled trial comparing continuous ESPB versus ICNB in patients undergoing VATS.^26^

Our results are consistent with Fiorelli *et al*.^27^, who reported lower opioid consumption in the first 48 hours after surgery in the ESPB group compared to the ICNB group. However, they reported higher MIP and MEP in the ESPB group. Our results are inconsistent with Turhan *et al*., who reported significantly lower opioid consumption in the ICNB than the ESPB group, but they observed only single-shot ESPB.^24^

The mean piritramide use between the groups started to differentiate after 12 hours postoperatively, marking the time when the ICNB effect wears out. Other observed parameters, such as time to chest tube removal, hospital discharge, and complications were similar in both groups. However, continuous ESPB is more demanding from the catheter insertion procedure to regular post-operative observations.^28^ The main advantages of ESPB presumably come from prolonged local anesthetic administration, enabling individual adjustments according to NRS scores.^29^

Despite the continuous neuromuscular block of the hemi-thoracic musculature, the ability to fully perform respiratory physiotherapy was not compromised by the continuous ESPB. MIP and MEP measurements decreased substantially but did not differ significantly between the two groups after 24 hours or after 48 hours. Because the absolute MIP and MEP results vary strongly in literature, we included relative values with the patients’ pre-operative measurements as the baseline.^30,31,32^

As common complications after lung resection, cardiac arrhythmias were monitored.^33^ Clinicians detected no arrhythmias as a consequence of local anesthetic administration, showing there were no cases of local anesthetic cardiotoxicity. The observed arrhythmias emerged later and were attributed to other post-operative factors.

Comparing ESPB to a placebo without regional anesthesia would be unethical considering the benefits of regional truncal blocks that have already been proven.^34^ The ICNB's efficacy has been assessed in a large meta-analysis of 66 eligible studies.^35^ The ICNB is reported to be superior to systemic analgesia, non-inferior to thoracic-epidural anesthesia, and marginally inferior to paravertebral block in the first 24 hours after surgery. The data suggests that the analgetic benefit of the ICNB slowly vanishes in 24 to 48 hours after surgery. Therefore, it is a reasonable comparison in 48 hours after surgery.

The main limitation of the study is the inability to double-blind the analgesic method because of the catheter. The second limitation is the number of included patients. It would be reasonable to conduct another study on a larger scale to see whether any other inter-group differences appear. The third limitation is the protocol for the continuous ESPB, which is subject to future changes regarding local anesthetic selection and administered volume. Ropivacaine is currently the best local anesthetic of choice because of its large safety profile and the lowest potential risk for cardiotoxicity^36^, while new anesthetics such as liposomal bupivacaine are being researched.^37^

## Conclusions

The study in our institution showed that the continuous ESPB decreases total opioid consumption and subjective pain perception at rest in the first 48 hours after VATS lung tumor resection compared to the intrathoracic ICNB. On the other hand, ESPB demands more nursing care. Regarding time to chest tube removal, hospital discharge, NRS values at cough and respiratory muscle strength, there were no observed differences between ICNB and ESPB.
